# Natural Selection Footprints Among African Chicken Breeds and Village Ecotypes

**DOI:** 10.3389/fgene.2019.00376

**Published:** 2019-05-08

**Authors:** Ahmed R. Elbeltagy, Francesca Bertolini, Damarius S. Fleming, Angelica Van Goor, Chris M. Ashwell, Carl J. Schmidt, Donald R. Kugonza, Susan J. Lamont, Max. F. Rothschild

**Affiliations:** ^1^Department of Animal Science, Iowa State University, Ames, IA, United States; ^2^Department of Animal Biotechnology, Animal Production Research Institute, Giza, Egypt; ^3^Virus and Prion Diseases of Livestock Research Unit, National Animal Disease Center, Agricultural Research Service, United States Department of Agriculture, Ames, IA, United States; ^4^Institute of Food Production and Sustainability, National Institute of Food and Agriculture, United States Department of Agriculture, Washington, DC, United States; ^5^Department of Poultry Science, North Carolina State University, Raleigh, NC, United States; ^6^Department of Animal and Food Sciences, University of Delaware, Newark, DE, United States; ^7^Department of Agricultural Production, Makerere University, Kampala, Uganda

**Keywords:** selection signatures, environmental stresses, African chicken, *F_ST_*, runs of homozygosity

## Abstract

Natural selection is likely a major factor in shaping genomic variation of the African indigenous rural chicken, driving the development of genetic footprints. Selection footprints are expected to be associated with adaptation to locally prevailing environmental stressors, which may include diverse factors as high altitude, disease resistance, poor nutrition, oxidative and heat stresses. To determine the existence of a selection footprint, 268 birds were randomly sampled from three indigenous ecotypes from East Africa (Rwanda and Uganda) and North Africa (Baladi), and two registered Egyptian breeds (Dandarawi and Fayoumi). Samples were genotyped using the chicken Affymetrix 600K Axiom^®^ Array. A total of 494,332 SNPs were utilized in the downstream analysis after implementing quality control measures. The intra-population runs of homozygosity (ROH) that occurred in >50% of individuals of an ecotype or in >75% of a breed were studied. To identify inter-population differentiation due to genetic structure, *F_ST_* was calculated for North- vs. East-African populations and Baladi and Fayoumi vs. Dandarawi for overlapping windows (500 kb with a step-size of 250 kb). The ROH and *F_ST_* mapping detected several selective sweeps on different autosomes. Results reflected selection footprints of the environmental stresses, breed behavior, and management. Intra-population ROH of the Egyptian chickens showed selection footprints bearing genes for adaptation to heat, solar radiation, ion transport and immunity. The high-altitude-adapted East-African populations’ ROH showed a selection signature with genes for angiogenesis, oxygen-heme binding and transport. The *neuroglobin* gene (GO:0019825 and GO:0015671) was detected on a Chromosome 5 ROH of Rwanda–Uganda ecotypes. The sodium-dependent noradrenaline transporter, *SLC6A2* on a Chromosome 11 ROH in Fayoumi breed may reflect its active behavior. Inter-population *F_ST_* among Egyptian populations reflected genetic mechanisms for the Fayoumi resistance to Newcastle Disease Virus (NDV), while *F_ST_* between Egyptian and Rwanda–Uganda populations indicated the Secreted frizzled related protein 2, *SFRP2*, (GO:0009314) on Chromosome 4, that contributes to melanogenic activity and most likely enhances the Dandarawi chicken adaptation to high-intensity of solar radiation in Southern Egypt. These results enhance our understanding of the natural selection forces role in shaping genomic structure for adaptation to the stressful African conditions.

## Introduction

Chicken domestication began in Asia as a combination of several local domestication events between 6,000 and 8,000 years ago (Miao et al., 2013; Mwacharo et al., 2013). Meanwhile, intensive human-directed selection for economic traits and the development of breeds is much more recent. A study based on mitochondrial D-loop sequences (Osman et al., 2016) suggested that African chickens can be separated into two clades: the first includes North-African (e.g., Egypt), Central African, European, and West and Central Asian chickens, while the second clade includes East-African (e.g., Uganda and Rwanda) and the Pacific chickens. The authors suggested that the first clade group likely originated from South-Asia and migrated to West-Asia, then arrived in Africa through Egypt, while the second clade migrated from the Pacific to East-Africa through the Indian Ocean. Present Egyptian chicken populations, as an example of the North-Africa chickens, include pure native breeds, such as Fayoumi and Dandarawi, and admixed fowl ecotypes which originated from unplanned crossings among native populations and are identified by their geographic distribution (ecotypes), such as the Baladi (synonym of local) and its naked neck type (Hosny, 2006). The Fayoumi is a medium-sized breed (average 2 kg for male and 1.6 kg for female) characterized by early maturation (150 days), aggressive behavior, flying ability and resistance to several pathogens, including resistance to Rous Sarcoma (Prince, 1958), Marek’s disease virus (Lamont et al., 1996) and *E. tenella* infection (coccidiosis) (Pinard-van der Laan et al., 2009; Bacciu et al., 2014). The Dandarawi is an auto-sexing bird and the smallest Egyptian breed (average 1.4 kg for male and 1.2 kg for female). This breed originated in Southern Egypt (Qena Governorate) which is characterized by hot (>40°C) dry climate, with intensive solar radiation. In Uganda and Rwanda, representing East Africa, where chicken breeding programs are absent, there are different admixed chickens (ecotypes) that vary in phenotypic characteristics and performance (Fleming et al., 2016).

According to the Koppen climate classification (Peel et al., 2007), Egypt is located in the Warm desert climate zone, while Uganda and Rwanda are in the Tropical savanna zone. The main environmental differences between Egypt and both Eastern Africa countries are altitude, precipitation, and temperature. According to the World Meteorological Organization (WMO), World Weather Information Service^1^, the 30-year averages for the major meteorological parameters for the capital of each country are as follows: Egypt has the hottest and driest weather with larger diurnal variation. Average temperatures ranged between 18.9 and 34.7°C and 2.47 ml of average annual precipitation rate. In Rwanda, average temperatures ranged from 25.9 to 28.2°C with an average annual precipitation rate of 79.24 ml, while Ugandan average temperatures ranged between 26.9 and 29.3°C with a precipitation rate of 105.24 ml. Altitude averages are, respectively, 75, 1,497 and 1,155 m in Egypt, Rwanda, and Uganda. For climatic variation among sampling locations of indigenous Egyptian chicken populations, Khalil et al. (2011) classified Egypt into six Agro-climatic zones according to the evapotranspiration (ETo) which considers major weather parameters, i.e., solar radiation, air temperature and humidity, and wind speed. According to the ETo mapping, Qalyubia (source of Baladi), Fayoum (source of Fayoumi) and Qena (source of Dandarawi) governorates belong to different ETo zones. The solar Atlas of Egypt (Khalil et al., 2010) indicated that average annual solar radiation ranges from 2,000 (North) to 3,200 (South) kWh/m^2^/year, and accordingly, Egypt was classified into 12 belts (zones). The Nile delta (including Qalyubia Governorate, source of Baladi ecotype) is located in a solar radiation belt that receives between 5.5 and 6.6 kWh/m^2^/day, while Fayoum (Mid-Egypt) receives 7.0–7.3 kWh/m^2^/day and Qena (Southern Egypt and source of Dandarawi) receives 8.3–8.5 kWh/m^2^/day. For solar radiation estimates in Rwanda, Batalla and Parellada (2015) reported a much lower variation than Egypt that ranged between 4.98 kWh/m^2^/day in Kayonza district and 5.28 kWh/m^2^/day in Bugesera district. While annual ETo (mm/day) varied between 4.49 in Kayonza and 4.9 in Bugesera districts. In Uganda, average solar radiation ranged between 17.2 MJ/m^2^ (4.78 kW/m^2^/day) in Kabale and 21.5 NJ/m^2^ (5.97 kWh/m^2^/day) in Soroti (Djaman et al., 2017). Under such wide spectrum of environmental variability in Egypt, which does not exist in Rwanda and Uganda, and absence of structural breeding plans, we speculate that rural chicken populations, in the study, are under different selection pressures driven by environmental stressors.

The current study aims to identify genomic footprints of natural selection of some North- vs. East-African chicken breeds and ecotypes raised and adapted to different local environments. The analytical approach combined high-density genotype-based, intra-population runs of homozygosity (ROH) and the allele-frequency-based inter-population genetic differentiation (*F_ST_*). ROH exist when identical haplotypes are inherited from each parent. ROH analysis indicated the population history and trait architecture (Ceballos et al., 2018). The length of ROH reflects individual demographic history and level of inbreeding. Meanwhile, the homozygosity burden can be used to detect genetic architecture of complex traits (Ceballos et al., 2018). It was also reported that ROH are universally common in genomes, even among outbred individuals of human. In cattle, a large proportion of ROH are likely the result of the accumulation of elite alleles from long-term selective breeding programs (Zhang et al., 2015). Therefore ROH was selected for studying population architecture and investigating selection signature resulted from natural selective forces in the indigenous African chicken breeds that are usually outbred and have been exposed to local natural selection forces for uncountable generations. *F_ST_* is one of the most widely used measures for assessing genetic differentiation. It plays a major role in ecological and evolutionary genetic studies. Since the emergence of next generation sequencing data, it was proved that the large number of genetic markers can compensate for small sample sizes when estimating *F_ST_* (Willing et al., 2012). With the variation in sample size among different chicken populations studied, *F_ST_* was selected for assessing genetic variation and detecting of inter-population selection signature.

## Materials and Methods

### Sample Collection, Genotyping, and Quality Control

A total of 268 blood samples were collected on FTA cards from birds of East Africa (EA; Rwanda and Uganda), and North Africa (NA; Egypt). Samples were collected by local veterinarians following the approved country standards of animal care practices. A total of 172 samples were collected in EA: 100 Rwandan and 72 Ugandan ecotypes. Rwandan samples were collected from the Huye (*n* = 25), Kicukiro, Kirehe, Musanze, Nyagatare, and Rubavu (*n* = 15 for each) districts. Ugandan samples were collected from three districts; Kamuli, Masaka, and Luweero (*n* = 24, for each). For more details on Ugandan and Rwandan samples see Fleming et al. (2016). A total of 96 samples were collected from Egypt: 31 Egyptian Native Naked Neck Baladi (will be referenced to as Baladi) from three villages in Qalyubia Governorate (30° 24′ 36″ N, 31° 12′ 36″ E, 19m) in the Delta; 31 Fayoumi from four villages in Mid-Egypt (Fayoum Governorate, 29° 21′ 48″ N, 30° 44′ 45″ E, 14m); and 34 Dandarawi from four villages in Southern Egypt (Qena Governorate, 26° 8′ 34.8″ N, 32° 43′ 40.8″ E, 76m). Chicken blood samples from Egypt, Rwanda, and Uganda were collected in accordance with the local veterinary guidelines in each country. All samples were collected with the consent of the owners of the chickens.

Genotyping of all samples was conducted at GeneSeek (Lincoln, NE, United States) using the Affymetrix Axiom^®^ 600k Array (Kranis et al., 2013). A total number of 494,332 SNPs and 266 birds were utilized in the downstream analysis after QC measures of MAF >0.05 and call rate of >0.97 applied to all samples using PLINK 1.9 (Chang et al., 2015). The raw data supporting the conclusions of this manuscript will be made available by the authors, on request, without undue reservation, to any qualified researcher.

#### Population Stratification and Structure

PLINK 1.9 (Chang et al., 2015) was used for constructing a multi-dimension scaling (MDS) plot based on a 266 × 266 matrix of genome-wide Identity-By-State (IBS) scores calculated based on pairwise comparisons of the genetic distances for all individuals, and the first two components. Ancestral model-based clustering, with no prior knowledge on breed origins, was performed using ADMIXTURE 1.2.2 (Alexander et al., 2009) to investigate individual admixture proportions, for 1 < *k* < 10, where *k* is the number of expected subpopulations, and the best *k* was determined based on the cross-validation error for different numbers of ancestral genetic backgrounds.

#### Runs of Homozygosity

Runs of homozygosity analyses were carried out for both individual populations and combined EA and NA breeds/ecotypes using PLINK 1.9 to examine overlapping genomic regions that harbored alleles driven to fixation within each population or group of populations using a SNP based sliding window approach. ROH requirements were defined as ≥300 SNPs, a minimum SNP density per ROH was set to one SNP per 50 kb, a maximum gap permitted between consecutive homozygous SNPs was set to 10 kb, three heterozygous calls were allowed within a run to account for genotyping errors and/or hitch-hiking events, and allelic match threshold of 0.95 identity and >20 SNPs. The overlapping ROH was considered as those overlapped across all populations, regardless their length, and consensus ROHs are those reached a consensus in either >50% of the individuals of an ecotype or in >75% of a breed, except for the Rwanda and Uganda ecotypes where a 40% consensus threshold was accepted. A gene ontology (GO) enrichment analysis was conducted for the list of genes located at the identified ROH consensus regions.

#### Fixation Index, *F_ST_*, for Inter-Breed Genetic Differentiation

To identify the regions under selection that are differentiated among breeds or ecotypes, an overlapping sliding window-based *F_ST_* analysis was calculated according to Karlsson et al. (2007). The pairwise comparisons were performed for North-African (Baladi, Dandarawi, and Fayoumi) vs. East-African (Rwanda and Uganda) populations, and all population-pairwise combinations, for overlapping windows along each chromosome. Each *F_ST_* window consisted of 500 kb with a step size of 250 kb. Only windows with ≥20 SNP were considered. Candidate genomic regions under selection were defined by a cutoff *F_ST_* value >0.30, that exceeds the value of 0.25 defined as very great genetic differentiation according to Hartl and Clark (1997). The GO enrichment analysis was also conducted on those genes located at the identified *F_ST_* windows.

#### Annotation and Enrichment

Genes within the regions of high interest for both ROH and *F_ST_* analyses were identified using the software bedtools v2.26.0 using the (*Gallus_gallus*-5.0, GCA_000002315.3) annotation genome^2^. GO for molecular function and biological processes for the identified genes were determined by PANTHER using the *Gallus gallus* reference genome^3^ and enriched genes were identified using Enrichr (Chen et al., 2013). GO terms were considered statistically significant at adjusted *P* < 0.05. Results were filtered using REVIGO^4^ (Supek et al., 2011), for removing redundancy to best classify significant GO terms per biological function.

## Results

### Population Stratification

**FIGURE 1:**
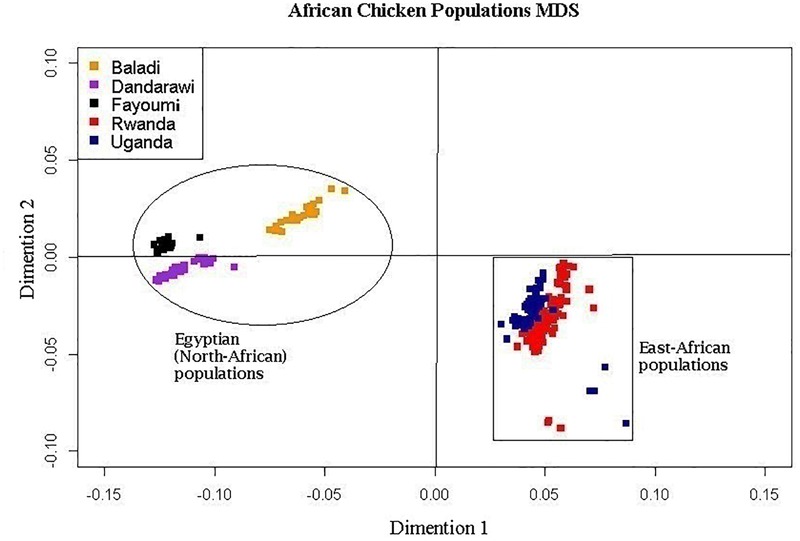
Multi-dimensional scaling, MDS, plot showing the distinct sampled five native African (two East- and three North-African) chicken populations. Plot was constructed based on a matrix of genome-wide Identity-By-State scores calculated based on pairwise comparisons of the genetic distances for all individuals. African chicken populations are Baladi ecotype (*N* = 31), Dandarawi breed (*N* = 33), Fayoumi breed (*N* = 30), Rwanda ecotype (*N* = 100), and Uganda ecotype (*N* = 72).

**FIGURE 2:**
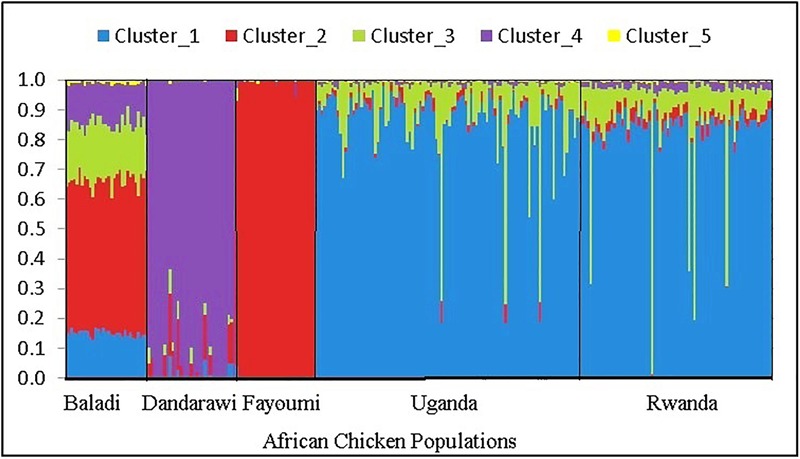
Admixture analysis plot for the five native African chicken populations, based on ancestral model-clustering, with no prior knowledge on breed origins. The optimum number of clusters (ancestral genetic background) *k* = 5. The five African chicken populations from left to right are three North-African (Baladi; *N* = 31; Dandarawi, *N* = 33; and Fayoumi, *N* = 30) and two East-African (Rwanda, *N* = 100 and Uganda, *N* = 72) populations.

The multi-dimensional scaling analysis (Figure 1) showed clear stratification and distinctive separation among the five populations studied. The first dimension (C1) separated the Egyptian (North-African) from both the Rwanda and Uganda (East-African) populations. The second dimension (C2) separated the Dandarawi (smallest-sized and tolerant to Southern Egypt extreme heat and solar radiation conditions) from both the Baladi (Nile Delta) and Fayoumi (Mid-Egypt). Baladi and Fayoumi (prevalent in similar environments of the Nile delta and Mid-Egypt) are genetically closer to each other than the Southern-Egypt Dandarawi breed. MDS also shows overlapping between the Rwandan and Ugandan populations, which was also reported by Fleming et al. (2016). For the admixture analysis, the best *K* (*K* = 5) was determined based on the cross-validation error for different numbers of ancestral genetic backgrounds. Admixture analysis (Figure 2) showed that Dandarawi and Fayoumi was the only population with minimal admixture. Baladi, Rwanda and Uganda are all ecotypes composed of an admixture of genetic backgrounds. Both Rwanda and Uganda chickens showed a composition of a one common main genetic background (ancestral genotypes) and four other minor backgrounds. Each of the ecotypes (Baladi, Rwanda, and Uganda) shares one of its minor genetic backgrounds with each of the Dandarawi and Fayoumi.

### Runs of Homozygosity (ROH) Mapping

Total individual ROH, regardless of consensus conditions, were classified according to length into three classes (Supplementary Table S1); short (300 kb–<1 Mb), medium (1–<1.5 Mb), and long (>1.5 Mb). The number and length of individual ROH differed widely among the populations in the study due to the nature of the population; e.g., breed or ecotype, number of samples and genetic structure. Breeds (Fayoumi and Dandarawi) showed higher average number of ROH than ecotypes. Egyptian Dandarawi showed the highest average number of ROH (180.8) and the highest percentage of medium (7.56%) and long (3.80%) ROH (Supplementary Table S1), indicating recent ancestral relationships and probably the highest inbreeding. For ecotypes, the Egyptian Baladi showed the lowest average number of ROH, and lowest number of long and medium-length ROH. This likely reflects ancestral relationships, low levels of inbreeding, a wider population gene pool, and geographical distribution in addition to genetic admixture.

### Intra-Population Footprints of Divergent Selection (Consensus ROH)

A total of 153 within-population consensuses ROH were detected with 41, 49, 35, and 28 in Baladi, Dandarawi, Fayoumi, and Rwanda–Uganda populations, respectively. Consensus ROHs were found on Chromosomes 3, 5, and 8 in Rwanda–Uganda; 2, 3, 4, 8, and 11 in Fayoumi; 1, 4, and 8 in Dandarawi; and 2, 3, 8, and 11 in Baladi (Supplementary Figure S1). The number of genes enriched and annotated within the overlapping consensus ROH was 62, 33, 72, and 29 genes for Baladi, Dandarawi, Fayoumi, and Rwanda–Uganda populations, respectively. The genes’ contribution to adaptation/tolerance performance is through their involvement in enzymatic (alpha amylase) and hormonal [corticosteroid and norepinephrine (NE)] activities; metabolism (lipid metabolism); reduction of oxidative stress (e.g., glutathione-*S*-transferase); tolerance to solar radiation (melanogenesis); ion binding and transport (sodium, potassium, and zinc); immunity and defense response (e.g., phagocytosis); oxygen-heme binding and transport; angiogenesis; apoptosis; tissue morphogenesis (e.g., bone trabecula formation); and tolerating acute heat stress (heat shock protein transcription factor).

### Signature of Selection Detected by ROH Mapping

The total 196 genes located on the consensus 153 ROH regions were used for detecting over-enriched GO terms. Enriched GO terms indicated biological processes and molecular functions promoting different mechanisms for adaptation to various cellular and environmental stressors (Table 1).

**Table 1:** A subset^1^ of gene ontology (GO) enrichment of consensus ROH analysis, and annotated genes in (a) East- and North-African populations, (b) East-African populations, and (c) North-African populations.

GO:Term	GO:ID	Genes
***(a) East- (Rwanda–Uganda) and North-African (Dandarawi and Fayoumi) populations***		
Alpha-amylase activity	GO:0004556	*AMY2A*
Calcium ion binding	GO:0005509	*SLC25A24*
Cellular response to oxidative stress	GO:0034599	*SLC25A24*
***(b) East-African (Rwanda–Uganda) populations***		
Angiogenesis	GO:0001525	*VASH1, Ang, ANGPT-1, ANGPT-2B, PGF*
Glutathione transferase activity	GO:0004364	*GSTZ1*
Heme binding.	GO:0020037	*NGB*
Oxygen binding	GO:0019825	*NGB*
Oxygen transport	GO:0015671	*NGB*
Regulation of lymph-angiogenesis	GO:1901491	*VASH1*
Response to wounding	GO:0009611	*VASH1*
***(c) North-African populations***		
**1. Dandarawi and Fayoumi**		
Chloride channel activities	GO:0005254	*CLCC1*
Chloride transmembrane transport	GO:1902476	*CLCC1, SLC12A3, SLC12A4, GLRA2*
**2. Baladi and Fayoumi**		
Dopamine uptake involved in synaptic transmission.	GO:0051583	*SLC6A2, PARK7*
Norepinephrine (NE) transport	GO:0015874	*SLC6A2*
Oxidoreductase activity	GO:0016491	*HSD11B2*
**3. Fayoumi**		
11-B hydroxysteroid dehydrogenase [NAD(P+)] activity	GO:0003845	*HSD11B2, HSD11B1a*
Anion transmembrane transport	GO:0098656	*SLC12A3, SLC12A4, CLCC1, GLRA2, SLC38A7*
Anion transport	GO:0006820	*SLC12A3, SLC12A4, SLC25A24, CLCC1, GLRA2, SLC10A2, SLC38A7, SERINC1, SLC38A8*
Bone trabecula formation	GO:0060346	*MMP2, SFRP1, FBN2*
Glucocorticoid biosynthetic	GO:0006704	*HSD11B2*
Growth factor activity	GO:0008083	*OSGIN1*
Regulation of apoptotic process	GO:0043065	*OSGIN1*
Regulation of bone remodeling	GO:0046850	*MC4R, TNFRSF11B*
Response to glucocorticoid	GO:0051384	*HSD11B2*
Skeletal system development	GO:0001501	*TRAPPC2, EXT1, DLX6, TRPS1, TNFRSF11B*
**4. Dandarawi**		
Melatonin receptor activity	GO:0008502	*Mel1c (GPR50), MTNR1A, MTNR1B, MTNR1C*
Response to radiation	GO:0009314	*SFRP2, THBD, CASP3, NPHP1, SDF4, NPHP4, ATM, ERCC5*
**5. Baladi**		
Protein homotrimerization	GO:0070207	*HSF1, HSF2, HSF3, HSF4*

*^*1*^The subset of GO that affect adaptation profile to cellular or environmental stressors and showed to be statistically significant.*

#### (a) Selection Signatures Common in East-African (Rwanda–Uganda) and North-African (Fayoumi and Dandarawi) Populations

Genes annotated within ROH and enriched GO terms reflected a common signature of selection for energy generation and transport; and ion binding in both the East-African (Rwanda–Uganda) and North-African (Fayoumi and Dandarawi) chicken populations studied. The (GO:0004556); molecular function of alpha-amylase activity was enriched and the *AMY2A* (alpha amylase2) gene (located on Chromosome 8) was annotated in the three African populations (Table 1). *AMY2A* is involved in the biological process of carbohydrates and glycogen metabolism, indicating the selection forces for metabolism, energy availability and response to thermal stress. Molecular function of calcium binding (GO:0005509) was commonly enriched in the same three populations. The annotated *SLC25A24* (solute carrier family 25 member 24, calcium-regulated mitochondrial ATP-Mg/Pi carrier), Chromosome 8, in both Rwanda–Uganda and Dandarawi (Table 1) is involved in the molecular function of calcium ion binding and energy (ATP) transmembrane transport. The (GO:0034599), physiological process of cellular response to oxidative stress was also commonly enriched in the same populations, indicating common signature of selection for responses to oxidative stresses.

#### (b) Selection Signatures in the East-African Populations

According to the environmental conditions of the two East-African countries studied (Rwanda and Uganda), the major stresses on the local chicken populations were oxidative stress, which is a common denominator for other stresses; high-altitude accompanied with lower oxygen availability; and lack of vaccination and poor health care. GO terms for molecular function of Oxygen binding (GO:0019825) and heme binding (GO:0020037); and physiological process of angiogenesis (GO:0001525), and oxygen transport (GO:0015671), Table 1, reflected adaptation to lower oxygen availability due to high altitude. Annotated genes resulted from the ROH mapping included two associated genes on Chromosome 5; vasohibin-1 (*VASH1*) and neuroglobin (*NGB*). *VASH1* gene is involved in the biological processes of angiogenesis (GO:0001525), response to wounding (GO:0009611) and regulation of lymphangiogenesis (GO:1901491). Neuroglobin (*NGB*) gene is associated with molecular functions of oxygen binding to heme (GO:0019825) and transport (GO:0015671) which contributes to the adaptation to high altitude and lack of oxygen stresses.

For the adaptation to oxidative-stress, the annotated glutathione-*S*-transferase zeta 1 (*GSTZ1*) increases the glutathione-*S*-transferase activity (GO:0004364) and the molecular functions of glutathione metabolic process (GO:0006749), and therefore decreases lipid oxidation products (Blackburn et al., 2006) as response to oxidative stress. Glutathione-*S*-transferase is also involved in a functional hepatic GST-mediated detoxification for the feed-borne mycotoxins.

#### (c) Selection Signatures in the North-African Populations

##### In both Dandarawi and Fayoumi

Two GO terms associated with chloride transport were enriched being the chloride channel activities (GO:0005254) and the chloride transmembrane transport (GO:1902476). The chloride channel CLIC like 1 (*CLCC1*) gene, Chromosome 8, was annotated in both GO terms in Dandarawi and Fayoumi (Table 1). *CLCC1 is* expressed in different organelles, including the endoplasmic reticulum (ER), Golgi apparatus, and nucleus in testis, spleen, liver, kidney, heart, brain, and lungs (Nagasawa et al., 2001), and involved in the biological processes of cation–anion (chloride) transport. The loss of *CLCC1* leads to disruption of chloride anion homeostasis in the ER and therefore disruption of protein-folding capacity and ER stress (Jia et al., 2015).

##### In both Baladi and Fayoumi (originated from Delta and Mid-Egypt regions)

Gene ontology terms for biological processes of NE transport (GO:0015874) and dopamine uptake involved in synaptic transmission (GO:0051583); and molecular function of oxidoreductase activity (GO:0016491) were commonly enriched (Table 1). For both NE transport and dopamine uptake the sodium-dependent noradrenaline transporter; solute carrier family 6 member 2 (*SLC6A2*), Chromosome 11, was annotated (Table 1). *SLC6A2* is involved in NE transport and is associated with the pathophysiology of attention-deficit/hyperactivity disorder (ADHD) in children (Sengupta et al., 2012). Dopamine uptake involved in synaptic transmission indicates the directed movement of dopamine into a presynaptic neuron or glial cell, where dopamine is a catecholamine neurotransmitter and a metabolic precursor of noradrenaline and adrenaline. Dopamine level in plasma was found to be highly correlated with adaptation to cold and heat stresses (Felver-Gant et al., 2012). *SLC6A2* then may contribute to the high physical activity and adaptation to heat stress in both Egyptian populations. In addition, the hydroxysteroid 11-beta dehydrogenase (*HSD11B2*), Chromosome 11, annotated for the oxidoreductase activity (GO:0016491) is a microsomal enzyme complex that oxidizes the glucocorticoid cortisol to the inactive metabolite cortisone. This activity limits the impact of cortisol and would support immunity and defense response of Fayoumi and Baladi populations.

##### In Fayoumi

Gene ontology terms for physiological process of both anion transport (GO:0006820) and anion transmembrane transport (GO:0098656) were enriched. Common putative annotated genes for those GO terms were the Na^+^-Cl^-^ cotransporter solute carrier family 12 member 3 (*SLC12A3)* and the K^+^-Cl^-^ cotransporter (*SLC12A4)*, Chromosome 11. *SLC12A3* is a cotransporter in the kidney that is involved in sodium ion transport and chloride transmembrane transport. It re-absorbs sodium and chloride ions from the tubular fluid into the distal convoluted tubule cells of the nephron. *SLC12A4* exhibits chloride symporter activity, playing key roles in electrolyte movement across epithelia and in intracellular chloride homeostasis of neurons and muscle cells (Payne, 2012). Annotated ion-transport related genes reflected the signature of selection for homeostasis that promotes adaptation in the Egyptian Fayoumi breed.

Three glucocorticoid associated GO terms were enriched, being the molecular function of 11-B hydroxysteroid dehydrogenase activity (GO:0003845) and both physiological processes of glucocorticoid biosynthesis (GO:0006704) and response to glucocorticoid (GO:0051384). The *HSD11B2* was the commonly gene annotated on Chromosome 11 (Table 1), in the three GO terms. *HSD11B2*, as previously mentioned, is a microsomal enzyme complex that oxidizes the glucocorticoid cortisol to the inactive metabolite cortisone, which limits the impact of cortisol.

The enriched physiological processes GO term of bone trabecula formation (GO:0060346) is involved in Fayoumi bone and ligaments morphogenesis (Table 1). The Matrix metallopeptidase 2 (*MMP2*) gene (Chromosome 11), annotated in this GO terms, contributes to the biological process of tissue morphogenesis; e.g., collagen catabolism and bone trabecula formation. *MMP2* may therefore, contribute to the distinctive morphogenesis characteristics of Fayoumi. Both GO terms of growth factor activity (GO:0008083) and regulation of apoptotic process (GO:0043065) were enriched in Fayoumi and the *OSGIN1* (oxidative stress-induced growth inhibitor 1), Chromosome 11 (Table 1) was annotated for both terms. *OSGIN1* encodes an oxidative stress response protein that regulates cell death and apoptosis by inducing cytochrome c release from mitochondria (Ott et al., 2002). *OSGIN1* inhibits growth in several tissues, e.g., ovary, kidney and liver, due to different causes of stresses. The homozygous genotype of *OSGIN1* could function in the Fayoumi stress response, including suppression of proliferation and the induction of apoptosis under the Egyptian stressful conditions.

##### In Dandarawi

Natural selection forces of the extreme stressful environment in Southern Egypt include severe hot weather, high-intensity of solar radiation, and lack of vaccination and poor health care services. Effects of these selective forces were reflected in the enriched GO terms of molecular function of Melatonin receptor activity (GO:0008502), and response to radiation (GO:0009314). Expression of the annotated melatonin receptor type 1C (*Mel1c*), Table 1, was reported to be associated with light intensity (Li et al., 2013; Park et al., 2014). The high solar intensity of Qena; 8.3–8.5 kWh/m^2^/day (Khalil et al., 2010), the source of Dandarawi, could be the selection force that fixed the *Mel1c* homozygosity. On Chromosome 4, the secreted frizzled-related protein 2 (*SFRP2*) was annotated (GO:009314; response to radiation). *SFRP2* is involved in chicken embryogenesis; development of the neural system (brain tissue), muscles (myogenesis), and developing eyes particularly the pigmented layer of the retina and photoreceptors (Lin et al., 2007). *SFRP2* stimulates melanogenesis through microphthalmia-associated transcription factor and/or tyrosinase upregulation via β-catenin signaling.

##### In Baladi

The Baladi is the only naked neck population (ecotype) in this study. The physiological process of protein homotrimerization (GO:0070207) enriched in this breed reflected the homotrimerization of heat shock protein factor. Heat shock factor proteins 1, 2, 3, and 4 were annotated on Baladi Chromosome 11 (Table 1), reflecting the population’s adaptation to heat. Xie et al. (2014) reported that *HSF4* exhibits tissue-specific expression with preferential expression in heart, brain, skeletal muscle, and pancreas, with two alternatively spliced isoform *HSF4a* and *HSF4b*. *HSF4a* acts as an inhibitor, while *HSF4b* as an activator of tissue specific heat shock gene expression.

### Fixation Index, *F_ST_*, for Inter-Populations Genetic Differentiation

Population stratification analyses (Figure 1) and ROH results (Table 1) indicated three genetically differentiated chicken groups that were considered for fixation index (*F_ST_*) analysis: (1) Dandarawi, (2) Baladi and Fayoumi (1 and 2 represent North-African populations), and (3) Rwanda and Uganda (East-African). Two comparisons were conducted; East-African vs. North-African and Baladi and Fayoumi vs. Dandarawi populations.

**FIGURE 3:**
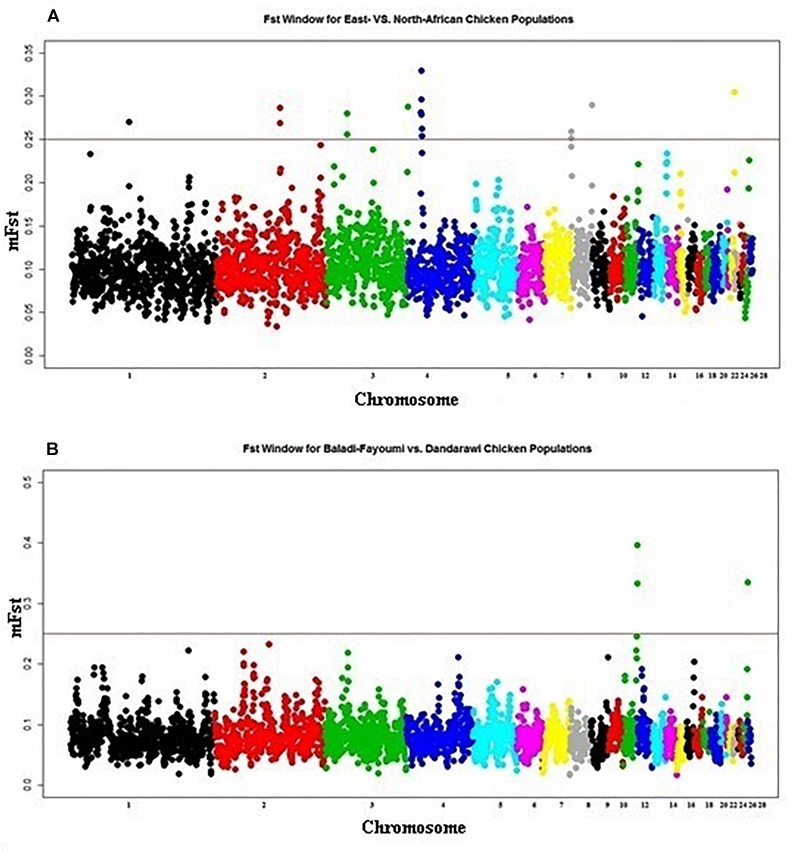
Manhattan plot for the *F_ST_* analysis of native African chickens populations showing the pairwise comparison of **(A)** East-African vs. North-African, and **(B)** Baladi–Fayoumi vs. Dandarawi populations. Plot is set based on *mF_ST_* (mean *F_ST_* across overlapping sliding windows). Vertical line presents cut-off threshold of *mF_ST_* = 0.25, representing very high genetic differentiation.

East-African vs. North-African *F_ST_* indicated one selection sweep on Chromosomes 4 (20.2–20.3 Mb), Figure 3A. Determined *F_ST_* regions, on Chromosome 4, indicated several genes playing roles in cell cycle, differentiation and proliferation, i.e., *SFRP2, FGA, FGB* and *FGG* (fibrinogen A, B, and G) and *PLRG1* (pleiotropic regulator 1). GO enrichment analysis indicated the GO term for physiological process of cell differentiation (GO:0030154). Within this enriched GO term, annotated genes were found to be contributing to the development of muscular and neural systems [myogenin (*MYOG*), *SFRP2*, neuropilin 1 (*NRP1*), and nerve growth factor (*NGF*)]. Myogenin (*MYOG*) acts as a transcriptional activator that promotes transcription of muscle-specific target genes and plays a role in muscle differentiation. *MYOG* induces myogenesis (fibroblasts to differentiate into myoblasts), in a variety of cells and tissues. The *SFRP2*, as previously indicated, is involved in chicken embryogenesis; development of the neural system (brain tissue), muscles (myogenesis), and developing eyes particularly the pigmented layer of the retina and photoreceptors (Lin et al., 2007). The *NRP1* is involved in the development of the cardiovascular system, angiogenesis, the formation of certain neuronal circuits and in organogenesis outside the nervous system. *NGF* is a neurotrophic factor and neuropeptide primarily involved in the regulation of growth, maintenance, proliferation, and survival of certain neurons. In addition, the annotated *MAPK9;* mitogen-activated protein kinase is involved in a wide variety of cellular processes such as proliferation, differentiation, transcription regulation and development. It targets specific transcription factors and mediates immediate-early gene expression in response to various cell stimuli, and is involved in UV radiation-induced apoptosis.

*Baladi and Fayoumi (Delta and Mid-Egypt) vs. Dandarawi (Southern Egypt) F_ST_* (Figure 3B) revealed two windows on Chromosome 11 (19.2–20.2 Mb). Functions of the annotated genes could explain some of the genetic variation among the Egyptian breeds focusing on the genetic uniqueness driven by extreme environmental stress and breeding practices in Southern Egypt (Dandarawi), and a distinctive immunity profile in Fayoumi. Enriched GO terms revealed the biological process and molecular functions associated with immunity, i.e., autophagy (GO:0006914) and positive regulation of natural killer cell mediated cytotoxicity (GO:0045954); regulation of skeletal muscle fiber development (GO:0048742); adaptation to oxidative stress, i.e., superoxide metabolic process (GO:0006801), cellular response to oxygen radical (GO:0071450) and nitric oxide biosynthetic process (GO:0006809); tolerance to irradiation and high-intensity of solar radiation, i.e., endosome to melanosome transport (GO:0035646) and melanosome organization (GO:0032438); and cell cycling and aging, i.e., regulation of telomere maintenance (GO:0032204) and negative regulation of telomere maintenance (GO:0032205).

The annotated putative genes also reflected the similar mechanisms of adaptation. For the immunity-relevant genes, the annotated GABA type A receptor associated protein like 2 (*GABARAPL2*), Table 2, is a member of the *Atg8* (autophagy-related protein 8) family that contributes to the formation of autophagosomes. This indicated genetic variation in autophagy process between the two genetic groups. For the cell cycle and bird aging associated gens, the *TERF2IP* (TERF2 interacting protein or telomeric repeat binding factor 2, RAP1) gene (GO:0032204 and GO:0032205) encodes a protein that is part of a complex involved in the biological processes of telomere length and protection (telomere maintenance, telomere maintenance via telomere lengthening and regulation of telomere maintenance).

**Table 2:** A subset^1^ of gene ontology (GO) enrichment and annotated genes within sweeps of inter-population *F_ST_*; (a) North- vs. East-African populations^2^ and (b) Baladi–Fayoumi vs. Dandarawi.

GO:Term	GO:ID	Genes
**(a) North- vs. East-African populations**		
Cell differentiation	GO:0030154	*SFRP2, PCK1, Pcdh15, NTRK3, MAPK9, NRP1, NGF, MYH9, MYL6, MYOG, MYBPC3*
**(b) Baladi–Fayoumi vs. Dandarawi**		
Autophagy	GO:0006914	*GABARAPL2, ATG5, VPS11*
Cellular response to oxygen radical	GO:0071450	*NQO1, SOD1, SOD2, SOD3, MNSOD, PRDX1*
Endosome to melanosome transport	GO:0035646	*AP1G1, RAB38, RAB32, ANKRD27, AP1M1*
Melanosome organization	GO:0032438	*AP1G1, HPS4, RAB38*
Negative regulation of telomere maintenance	GO:0032205	*TERF1, TERF2, TERF2IP, RTEL1, CTC1*
Nitric oxide biosynthetic process	GO:0006809	*NQO1, SLC7A2, NOS1, NOS2*
Positive regulation of natural killer cell mediated cytotoxicity	GO:0045954	*AP1G1, LAMP1, IL12B, IL12A, IL18RAP*
Regulation of skeletal muscle fiber development	GO:0048742	*ZFHX3, MYOG, MYF5, MYF6, MYOD1*
Regulation of telomere maintenance	GO:0032204	*TERF2IP, MRE11, TERF1, TERF2, TERF2IP, RTEL1, CTC1*
Superoxide metabolic process	GO:0006801	*NQO1, SOD1, SOD2, SOD3, NOS2, MNSOD*

*^*1*^The subset of GO that affect adaptation profile to cellular or environmental stressors. ^2^North-African populations group is composed of Baladi, Fayoumi, and Dandarawi populations’ samples; while East-African population group is composed of Rwanda and Uganda populations’ samples.*

The *AP1G1* (adaptor related protein complex 1 gamma 1 subunit) gene that was annotated in the two GO terms (GO:0035646 and GO:0032438) plays a major role in Dandarawi feather pigmentation (melanosome organization and transport). *AP1G1* could reflect the sex-linked variation in feather coloring (Dandarawi males and females have different colors) and Dandarawi tolerance to intensive solar radiation in Southern Egypt. *AP1G1* was also annotated in the GO:0045954 (positive regulation of natural killer), Table 2. *ZFHX3* (zinc finger homeobox 3) gene affects the regulation of myoblast differentiation and fiber development (GO:0048742; regulation of skeletal muscle fiber development).

For adaptation to oxidative stress, the annotated *SOD1*, superoxide dismutase [Cu–Zn] contributes to the biological processes of cellular response to Oxygen radicals (GO:0071450), and Superoxide metabolic process (GO:0006801), Table 2. *SOD1* role, as an anti-oxidizing enzyme, is to converts harmful superoxide radicals into less reactive oxygen species (ROS) and hydrogen peroxide, H_2_O_2_ (Bosco, 2015). In addition, the annotated *NQO1* [NAD(P)H dehydrogenase, quinone 1] is a major quinone reductases, that is highly inducible and plays multiple roles in cellular adaptation to stress. Reported roles of *NQO1* included its ability for quinone detoxification, to function as a component of the plasma membrane redox system generating antioxidant forms of ubiquinone and vitamin E, and its function as a superoxide reductase (Ross and Siegel, 2017).

Concerning the adaptation to thermal stress, the annotated *NOS1* (nitric-oxide synthase 1) contributes to the molecular function of nitric-oxide synthase activity (GO:0004517) and the nitric oxide biosynthetic process (GO:0006809). Yadav et al. (2016) demonstrated significantly higher expression of different types of NOS (*P* < 0.05) during summer and winter peaks, in goats, as compared to moderate season. Authors, therefore, indicated the possible involvement of the NOS family genes in ameliorating thermal stress and to maintain cellular integrity and homeostasis.

## Discussion

### Population Stratification

In African developing countries that lack genetic improvement schemes applied to indigenous chicken genetic resources, the major forces driving genetic diversity are natural biotic/abiotic stresses, including flock management. In this study, the MDS reflected genetic divergence between the smallest Egyptian breed, Dandarawi, adapted to Southern Egypt’s extreme heat and solar radiation conditions, from the other two populations belonging to a less stressful environment of Delta and Mid-Egypt. Both MDS and admixture analyses confirmed the genetic similarity between the Rwandan and Ugandan ecotypes that has been reported by Fleming et al. (2016). The admixture analysis confirmed that Baladi and Fayoumi share a major ancestral background. Gene flow from Fayoumi to indigenous Baladi ecotypes likely occurred as a result of indiscriminate breeding practices in the villages. East-African ecotypes (Rwanda and Uganda) share a portion of its genetic backgrounds with both the Dandarawi and Fayoumi breeds. Considering that (1) no genetic exchange was reported between the Egyptian and East-African populations, and (2) both North-African and East-African chickens belong to different clades (origins), according to the mitochondrial D-loop sequences study (Osman et al., 2016), such common genetic backgrounds could be due to the ancestral part of the genome. This may strengthen the hypothesis that ancient chickens were first introduced to Egypt, from Asia through the cinnamon trade and then transported to other parts of the African continent including Rwanda and Uganda (MacDonald, 1992; Blench and MacDonald, 2000; Mwacharo et al., 2013), a hypothesis that needs more investigation.

### Runs of Homozygosity (ROH) Mapping

A total of 153 within-population consensuses ROH were detected; 41, 49, 35, and 28 in Baladi, Dandarawi, Fayoumi, and Rwanda–Uganda populations, respectively. Chromosomal distribution of the within-population consensus ROH varied among the five populations studied, i.e., the highest ROH signals were found on Chromosomes 2, 3, 8 and 11 in Baladi; 2, 3, 4, 8, and 11 in Fayoumi; 1, 4, and 8 in Dandarawi; and 3, 5, and 8 in Rwanda–Uganda populations. Chromosome 8 was common among all studied populations in bearing signatures of selection. Fleming et al. (2016), studied ROH in Rwanda and Uganda populations, and reported different chromosomal distribution of overlapping and consensus ROH. This is due to the utilization of different ROH analysis parameters, overlapping, and consensus conditions. Fleming et al. (2016) considered overlapping ROH as those overlapped across all populations and contained 10 or more individuals and inter-breed consensus were those common to every bird, irrespective of length of the ROH.

### Signature of Selection Detected by ROH Mapping

#### (a) Selection Signatures Common in East- and North-African Populations

Under African village conditions, with lack of standardized rations, chicken feeding is mainly based on scavenging (free range), household waste and some grain supplementation. Therefore, carbohydrates metabolism, energy generation and transport are important traits for adaptation. The enriched GO term (GO:0004556; alpha-amylase activity) and the annotated *AMY2A* (alpha amylase 2) gene, in Rwanda–Uganda, Fayoumi and Dandarawi populations suggested the signatures of selection forces of carbohydrates and glycogen metabolism, and response to thermal stress and unbalanced feeding. On the same chromosome, the *SLC25A24* (solute carrier family 25 member 24, calcium-regulated mitochondrial ATP-Mg/Pi carrier) was annotated in both Rwanda–Uganda and Dandarawi populations. *SLC25A24* is involved in calcium ion binding (GO:0005509) and cellular response to oxidative stress (GO:0034599) (Ehmke et al., 2017; Harborne et al., 2017). *SLC25A24* imports adenine nucleotides from the cytosol into the mitochondrial matrix and exports phosphate to the cytosol. This process controls the size of the adenine nucleotide pool of the mitochondrial matrix in response to cellular energetic demands (Harborne et al., 2017) and supports the adenine-dependent mitochondrial activities including gluconeogenesis, mitochondrial biogenesis and mitochondrial DNA maintenance. Regulation of energy transport by *SLC25A24* is crucial for adaptation to stressful conditions in African villages. Fleming et al. (2016) reported over-enrichment of molecular functions of calcium ion binding (GO:0005509) as related to oxidative stress induced function by the environment, in East-African (Rwanda and Uganda) ecotypes. In the same study, authors also reported enriched GO:0034599 (cellular response to oxidative stress) in Rwanda ecotype.

#### (b) Selection Signature in the East-African Populations

In the absence of structured selection schemes for productive performance, stressful conditions are the major selection forces on indigenous East-African chicken populations. The stresses in the East-African countries include high-altitude and lower oxygen availability; and oxidative stress in addition to lack of vaccination and poor veterinary services. Altitude averages are 1,497 m and 1,155 m in Rwanda and Uganda, respectively, in comparison with 75 m in Egypt. High altitude is accompanied with lower partial oxygen pressure and less effective oxygen availability. Enriched GO terms indicated biological process of angiogenesis (GO:0001525), oxygen transport (GO:0015671); and molecular function of heme binding (GO:0020037), and oxygen binding (GO:0019825). Annotated genes within detected ROH in the Rwanda–Uganda populations reflected the effects of high altitude and management forces, e.g., feeding quality, on shaping genetic divergence. Vasohibin-1 (*VASH1*) gene is involved in angiogenesis, regulation of endothelial cell proliferation in response to wounding (GO:0009611), and regulation of lymphangiogenesis (GO:1901491) (Heishi et al., 2010; Miyashita et al., 2012; Affara et al., 2013; Sato, 2013). Fleming et al. (2017) also reported strong selection toward angiogenesis, and Fleming et al. (2016) reported the (GO:0042060; wound healing) in the Rwanda and Uganda populations. Neuroglobin, *NGB*, is a neuron-specific globin shown to protect against hypoxia, ischemia, oxidative stress and is associated with oxygen transport and oxygen-heme binding (Mammen et al., 2002; Milton et al., 2006; Hümmler et al., 2012). This reflected tolerance of Rwanda–Uganda chickens to high-altitude and wound healing. Oxidative stress resulted from various stressors, including heat, pathogen invasion, and high-solar radiation made oxidative stress a common denominator of stress responses in African chicken. The GO term of molecular function of glutathione transferase activity (GO:0004364) was enriched. *GSTZ1* (glutathione-*S*-transferase zeta 1) annotated in Rwanda–Uganda, enables the molecular functions of glutathione transferase and peroxidase activities in response to oxidative stress. Fleming et al. (2016) reported signatures of selection related to genes and signaling pathways involved in the reduction of ROS through utilization of calcium ions, lipids, and kinases, as the mobilization of Ca^2+^ is a part of the trade-off in Ca^2+^ usage between the calcium needed for eggshell formation and that stored in the ER. Maize contamination with the feed-borne mycotoxin aflatoxin B1 (AFB1) is a common problem in East-African humid environment. Nishimwe et al. (2017) reported that most of the animal feed containing maize has >100 μg/kg of AFB1 in Rwanda. AFB1 has a high hepatotoxic effect on different poultry species. It was found that domesticated turkeys (*Meleagris gallopavo*) was very susceptible to the AFB1 because it lacks a functional hepatic GST-mediated detoxification of AFBO (electrophilic exo-AFB1-8,9-epoxide), while the wild turkey (*Meleagris silvestris*) was resistant due to its hepatic ability for GST-mediated AFBO detoxification (Reed et al., 2018). Annotated *GSTZ1* could be reflecting natural selection for both reduction of oxidative stress and resistance to aflatoxins in Rwanda–Uganda ecotypes.

#### (c) Selection Signature in the North-African (Egyptian) Chicken Populations

A crucial factor in stress tolerance is the dynamic relationship between cations and anions to maintain body fluid and cell homeostasis (Mongin, 1980). Calcium, potassium, and sodium are major cations while chloride is a major anion in chicken. Low chloride levels can affect the acid–base balance and increase blood pH. Several GO terms associated with cation/anion binding and transport were enriched in Egyptian populations: Chloride channel activities (GO:0005254) and chloride transmembrane transport (GO:1902476) in Dandarawi and Fayoumi; anion transport (GO: 0006820) and anion transmembrane transport (GO: 0098656) in Fayoumi. Fleming et al. (2016) reported the enrichment of GO terms of calcium ion transmembrane transport (GO:0070588) in consensus ROH in Rwanda and Uganda populations, which may indicate the common contribution of anion/cation balance in adaptation profile of African chicken populations. Several genes associated with cation/anion binding and transport were annotated in the consensus ROH of the Egyptian breeds: The chloride channel CLIC like 1 (*CLCC1*), annotated in Fayoumi and Dandarawi; the Na^+^-Cl^-^ co-transporter solute carrier family 12 member 3 (*SLC12A3*) and the K^+^-Cl^-^ co-transporter solute carrier family 12 member 4 (*SLC12A4*) annotated in Fayoumi. *CLCC1* was reported by Nagasawa et al. (2001) to be expressed in different organelles, including the ER and kidney. Jia et al. (2015) proved that loss of *CLCC1* and disruption of chloride homeostasis in the ER disrupted the protein-folding capacity of the ER and resulted in ER stress, misfolded protein accumulation, and neurodegeneration. *SLC12A3* is a cotransporter in the kidney that re-absorbs sodium and chloride ions from the tubular fluid into the distal convoluted tubule cells of the nephron. *SLC12A4* plays key roles in electrolyte movement across epithelia and in intracellular chloride homeostasis of neurons and muscle cells (Payne, 2012). It was also reported to contribute to the osmotic fragility of erythrocytes (Hanzawa et al., 2002). Annotated ion-transport related genes reflected the signature of selection for homeostasis and metabolism that promoted stress tolerance in the Egyptian chicken populations.

In both Fayoumi and Baladi (sourced from Delta and Mid-Egypt and showed common ancestral background), results indicated that the adrenaline and noradrenaline play roles in their adaptation profiles, as both the biological processes of NE transport (GO:0015874) and dopamine uptake (GO:0051583) were enriched. Dopamine is a neurotransmitter and a precursor of adrenaline. Stress activates the hypothalamus–pituitary–adrenal (HPA) axis, which increases the release of glucocorticoids from the adrenal glands that in concert with other neuro-modulators, e.g., noradrenaline, promote cognitive adaptation to stressful conditions (Krugers et al., 2012). Sodium-dependent noradrenaline transporter (solute carrier family 6 member 2, *SLC6A2*) was annotated in both Fayoumi and Baladi, while 11β-hydroxysteroid dehydrogenase type 2 (*HSD11B2*) was annotated in Fayoumi. *SLC6A2* is involved in NE transport and availability, while *HSD11B2* oxidizes the glucocorticoid cortisol to the inactive metabolite cortisone, preventing illicit activation of the mineralocorticoid receptor. *HSD11B2* is expressed in aldosterone-sensitive neurons and responsible for promoting appetite for sodium (feeding behavior), independently of thirst or hunger (Jarvie and Palmiter, 2017). Inhibition of *HSD11B2* causes mineralocorticoid excess and hypertension due to inappropriate glucocorticoid activation of renal mineralocorticoid receptors (Chapman et al., 2013). *HSD11B2* oxidization of the glucocorticoids would support immunity and defense response of Fayoumi (Prince, 1958; Lamont et al., 1996; Pinard-van der Laan et al., 2009; Bacciu et al., 2014).

Fayoumi is characterized with ability to fly which is expected to be reflected in their genome structure and selection footprints. Two GO terms of biological processes of bone trabeculae formation (GO:0060346) and regulation of bone remodeling (GO:0046850) were enriched in Fayoumi. *MMP2* (matrix metallopeptidase 2) annotated in Fayoumi contributes to the biological process of tissue morphogenesis, collagen catabolism and bone trabecula formation (spongy bone that contains the red bone-marrow). The annotated *TNFRSF11B*, is a member of the TNF-receptor superfamily, which is responsible for the production of an osteoblast-secreted decoy receptor that functions as a negative regulator of bone resorption. Both annotated *MMP2* and *TNFRSF11B* may be related to distinctive morphogenesis characteristic of mineral density and ability to fly in Fayoumi (Geleta et al., 2013).

Oxidative stress increases levels of lipid peroxidation along with elevating hydrogen peroxide levels in the cytosol and mitochondria (Chandrashekar and Muralidhara, 2010). To offset oxidative stress, cells respond with elevated glutathione levels, increased activities of glutathione-dependent enzymes and increased membrane permeability and intracellular Ca^+^ levels. Multiple genes contributing to oxidative stress reduction were annotated; *OSGIN1* and *HSD11B2* (hydroxysteroid 11-beta dehydrogenase 2) in Fayoumi. *OSGIN1* encodes an oxidative stress response protein that regulates cell death and apoptosis by inducing cytochrome c release from mitochondria (Ott et al., 2002). *OSGIN1* expression is regulated by p53 and induced by DNA damage and inhibits growth in several tissues. The homozygous genotype of *OSGIN1* could play role in the Fayoumi response to oxidative stress, with anti-proliferative function and the induction of apoptosis at the cost of growth performance under village stressful conditions.

Selection forces of the severe stressful hot-dry and high solar intensity conditions in Southern Egypt showed their signature on the Dandarawi genome. The two GO terms of molecular function of melatonin receptor activity (GO:0008502) and the biological process of response to radiation (GO:0009314) were enriched. Expression of melatonin receptor type 1C (*Mel1c*; ortholog of mammalian *GPR50*), is activated by monochromic light (green light) in several organs, and subsequently, activates several immune- and developmental-related processes within these organs. For instance, *Mel1c* activates B-lymphocyte proliferation in broiler bursa (Li et al., 2013), T-lymphocyte proliferation in broiler thymus (Chen et al., 2016), development of the newly hatched chick’s liver via an anti-oxidation pathway (Wang et al., 2014) and secretion of insulin-like growth factor 1 in chicks embryo liver (Li et al., 2016). The high solar intensity of Qena, the source of Dandarawi; 8.3–8.5 kWh/m^2^/day (Khalil et al., 2010) could be the selection force that fixes the *Mel1c* homozygosity in the Dandarawi breed to promote its adaptation and immunity characteristic. As previously indicated, the annotated *SFRP2* stimulates melanogenesis through microphthalmia-associated transcription factor and/or tyrosinase upregulation via β-catenin signaling.

The Baladi ecotype has unique heat tolerance due to the naked neck phenotype, compared with the other populations in this study. The enriched GO of biological process of protein homotrimerization (GO:0070207) and the annotated heat shock transcription factors 1, 2, 3, and 4 (*HSF1, 2, 3*, and *4*) revealed the population adaptation to the Egyptian heat condition. The heat shock proteins are chaperone proteins that effectively protect several proteins and cell organelles from stressors’ negative effects, mainly heat. Heat shock transcription factors, e.g., *HSF4* exhibits tissue-specific expression with two alternatively spliced isoforms; *HSF4a* and *HSF4b*. *HSF4a* acts as an inhibitor, while *HSF4b* as an activator of tissue specific heat shock gene expression (Xie et al., 2014).

### Fixation Index, *F_ST_*, for Inter-Populations Genetic Differentiation

To study the genomic differentiation resulting from forces of natural environmental stresses, two scopes were proposed. First, genomic variation among North- vs. East-African chicken populations (hot arid desert vs. tropical Savana, according to Peel et al., 2007). The second is variation between Baladi and Fayoumi vs. Dandarawi, considering results of population stratification and similarity in ROH mapping between Baladi and Fayoumi. This allowed investigating both inter-population genomic variation and the possible signatures of selection due to climatic variation between delta/Mid Egypt and Southern-Egypt regions.

For the genomic differentiation resulting from selection forces of the distinct climates between North- and East-African countries studied, the GO term for biological process of cell differentiation (GO:0030154) was enriched. Multiple genes contributing to the development of muscular and neural systems were annotated (*SFRP2*, MAPK9, *MYOG, NRP1*, and *NGF*). The annotated *SFRP2*, as previously indicated, stimulates melanogenesis through microphthalmia-associated transcription factor and/or tyrosinase upregulation via β-catenin signaling (Kim et al., 2016). *MAPK9* (mitogen-activated protein kinase 9) is involved in a wide variety of cellular processes such as proliferation, differentiation, transcription regulation and development. It targets specific transcription factors and mediates immediate-early gene expression in response to various cell stimuli, and is involved in UV radiation-induced apoptosis. Annotated *SFRP2* and *MAPK9* reflected selection footprints for the high-intensity of solar radiation in Southern Egypt governorate; Qena (source of Dandarawi), that receives 8.3–8.5 kWh/m^2^/day of solar radiation (Khalil et al., 2010). Scavenging Dandarawi is highly affected by higher intensity of solar radiation, and melanogenic activity of the *SFRP2* very likely contributed to their adaptation. Phenotypic variations among North-African (Baladi, Dandarawi, and Fayoumi), and East-African (Rwanda and Uganda) populations were reflected in the annotated myogenin gene. Myogenin (*MYOG*) induces myogenesis (fibroblasts to differentiate into myoblasts), in a variety of cells and tissues, through its actions as a transcriptional activator that promotes transcription of muscle-specific target genes. Both *NRP1* and *NGF* genes play role in neuron development. The *NRP1* is involved in the development of the cardiovascular system, angiogenesis, the formation of certain neuronal circuits and in organogenesis outside the nervous system. *NGF* is a neurotrophic factor and neuropeptide primarily involved in the regulation of growth, maintenance, proliferation, and survival of certain neurons. Alteration in incubation conditions of developing chicks might change the developmental trajectories of some physiological regulation systems and may affect the quality of the young check during the first few days’ post-hatching (Tzschentke and Plagemann, 2006). Tong et al. (2013) reported that incubation conditions, embryonic physiological parameters, and other environmental factors are important for prober differentiation and actual hatching times. Environmental variation between North- and Eastern-African sampling locations (Egypt vs. Rwanda and Uganda) and their effects on chicks’ embryonic development and cell differentiation could be the selection forces for the annotated genes.

*F_ST_* analyses compared between Delta/Mid-Egypt populations (Baladi and Fayoumi) and the Southern Egypt (Dandarawi) revealed genetic variation resulting from the different environmental stresses and breeding practices in Southern Egypt. *Atg8* contributes to the formation of autophagosomes (Kabeya et al., 2000). The annotated GABA type A receptor associated protein like 2 (*GABARAPL2*) (GO:0006914; biological process of autophagy), is a member of this family. Sun et al. (2014) reported that Newcastle Disease Virus (NDV) triggers autophagy resulting in enhanced virus replication in chicken cells and tissues. Results of Hassan et al. (2004), based on the challenge of four Egyptian chicken breeds with NDV indicated that Dandarawi, along with the Gimmizah synthetic breed, were highly susceptible (100% mortality for both breeds) to NDV infection. Deist et al. (2017) reported that the Fayoumi showed a significantly less viral load than the Leghorns at 6 days-post-infection, indicating the Fayoumi potentiality to clearing the virus and possibly overcoming infection more efficiently than the Leghorns. The *F_ST_* results could reflect the variation between Fayoumi and Dandarawi in autophagy, which indicated variation in their resistance to NDV.

Under rural poultry production in Southern Egypt, no regular culling (for genetic improvement) is practiced and birds are prone to extended production life, associated with an extended number of cell cycles, which could promote a signature of selection for telomere length and stability. FAO (2009) in a study for characterizing the domestic chicken and duck production systems in Egypt indicated that 100% of the interviewed households (209 households) in Sohag (the Qena neighboring governorate) reported the longevity as a major criterion for selecting the birds that they buy. The enriched GO terms of biological processes of Negative regulation of telomere maintenance (GO:0032205) and regulation of telomere maintenance (GO:0032204) may reflect the variation in birds longevity between Egyptian populations under different production systems. *TERF2IP* gene, annotated in the two GO terms, encodes a protein that is part of a complex involved in telomere length and protection (O’Connor et al., 2004; Martinez et al., 2010; Chen et al., 2011). It is likely that the annotated *TERF2IP* was a signature of selection for “longevity” in Southern-Egypt Dandarawi breed.

Climatic variation among sampling locations of indigenous Egyptian chicken populations had been reported. Egypt was classified into 12 zones according to the solar Atlas of Egypt (Khalil et al., 2010). The Nile delta (source of Baladi) receives 5.5–6.6 kWh/m^2^/day; Fayoum (Mid-Egypt and source of Fayoumi population) receives 7.0–7.3 kWh/m^2^/day; and Qena (Southern Egypt and source of Dandarawi) receives 8.3–8.5 kWh/m^2^/day. With absence of structural breeding plans, we speculated that Egyptian rural chicken populations, in the study, are under different selection pressures driven by variations in solar radiation. The enriched GO terms for biological processes of endosome to melanosome transport (GO:0035646) and melanosome organization (GO:0032438) could emphasizes variation between Dandarawi and both Baladi and Fayoumi in their tolerance to solar radiation stress. The *AP1G1* (adaptor related protein complex 1 gamma 1 subunit) gene, annotated in both GO terms, plays a major role in feather pigmentation (melanosome organization and transport). *AP1G1* could reflect both the sex-linked variation in feather coloring (Dandarawi males and females have different colors), and tolerance to intensive solar radiation in Dandarawi under Southern Egypt stressful environment. Fleming et al. (2016) reported the enrichment of GO term for response to radiation (GO:0009314) and DNA repair (GO:0006281), in Rwanda and Uganda populations, justifying that as possibly a result of the birds living at the equator.

## Conclusion

In conclusion, results of this study indicated that environmental stresses played major roles in shaping genomic variation of African chicken populations. In Egypt, Baladi and Fayoumi were genetically closer to each other than the Southern-Egypt Dandarawi population, while Rwanda and Uganda chickens showed clear overlap in their genomic structure, being under very similar environmental conditions. Although, no genetic exchange was reported between Egyptian populations (Fayoumi and Dandarawi) and East-African ecotypes (Rwanda and Uganda), the existence of some common ancestral genetic backgrounds among the two groups of populations could be due to the ancestral part of the genome, according to the hypothesis that ancient chickens were first introduced to Egypt, from Asia through the Cinnamon trade and then transported to other parts of the African continent including Rwanda and Uganda.

Intra-population ROH and inter-population *F_ST_* mapping revealed selection footprints of possible environmental stresses, breed characteristics and management. ROH of all native African populations showed selection footprints for energy transport, calcium ion binding, and reduction of oxidative stress. North-African (Egyptian) populations, under hot desert environment, showed likely selection footprints for adaptation to heat, solar radiation, ion transport and immunity. East-African populations, under tropical savanna and higher altitude conditions, showed a signatures of selection for oxygen-heme binding and transport, and reduction of oxidative stress. Behavior and phenotypic characteristics were reflected by ROH mapping in the study. Genes associated with availability and transport of corticosteroid and NE could reflect the active behavior of the Fayoumi breed. *F_ST_* mapping and its annotated genes emphasized the genetic variations likely generated by natural selective forces. Egyptian Fayoumi showed distinctive genetic mechanisms for their resistant to the endemic diseases, e.g., NDV. Management issues of chicken flocks, including extended bird longevity in the Southern-Egypt households was also reflected in terms of genes associated with telomere maintenance. These results enhance our understanding of the role of natural selection forces in shaping genomic variation, and genes contributing to adaptation under stressful African conditions.

## Ethics Statement

The study presented in the manuscript involve blood sample collection from rural chicken in some Egyptian, Rwandan and Ugandan villages. Samples were collected by local villages veterinarians following the approved country standards for minimizing any probable bird uncomfortability.

## Author Contributions

AE, FB, and MR conceptualized and designed the work. DF, AVG, and DK collected the sample and data. AE, FB, SL, and MR analyzed and interpreted the data. AE drafted the manuscript. FB, DF, AVG, CA, CS, DK, SL, and MR made a critical revision of the manuscript. All authors have approved the final version of manuscript to be published.

## Conflict of Interest Statement

The authors declare that the research was conducted in the absence of any commercial or financial relationships that could be construed as a potential conflict of interest.

## Footnotes

1 http://worldweather.wmo.int/en/home.html

2 http://useast.ensembl.org/Gallus_gallus/Info/Index?db=core

3 http://www.pantherdb.org/

4 http://revigo.irb.hr/
