# Supplementary material for: Natural Selection Footprints Among African Chicken Breeds and Village Ecotypes
Source: Front Genet. 2019 May 8;10:376. doi: 10.3389/fgene.2019.00376 (PMC6518202; doi:10.3389/fgene.2019.00376)
Supplement: Supplementary file 1 [file Table_1.DOC]

**Supplementary Tables**

**Table (S1) ROH number (and %) of different lengths categories distribution in the five African chicken populations studied**

| **ROH length Category** | **Fayoumi**  **(N=30)** | **Dandarawi**  **(N=33)** | **Baladi**  **(N=31)** | **Rwanda**  **(N=100)** | **Uganda**  **(N=72)** | **Total**  **(N=266)** |
| --- | --- | --- | --- | --- | --- | --- |
| **Short (300kb-<1Mb)** | 4,441 (*91.64*) | 5,290 (*88.64*) | 1,603 (*96.74*) | 7,725 (*94.83*) | 5,606 (*90.68*) | **24,665** |
| **Medium (1Mb-<1.5Mb)** | 312 (*6.44*) | 451 (*7.56*) | 44 (*2.66*) | 314 (*3.85*) | 408 (*6.60*) | **1,529** |
| **Long (>1.5Mb)** | 93 (*1.92*) | 227 (*3.80*) | 10 (*0.60*) | 107 (*1.31*) | 168 (*2.72*) | **605** |
| **Total** | **4,846** | **5,968** | **1,657** | **8,146** | **6,182** | **26,799** |
| **Average No. ROH/sample** | **161.5** | **180.8** | **53.5** | **81.5** | **85.9** |  |
