# Supplementary figures and images for: Natural Selection Footprints Among African Chicken Breeds and Village Ecotypes
Source: Front Genet. 2019 May 8;10:376. doi: 10.3389/fgene.2019.00376 (PMC6518202; doi:10.3389/fgene.2019.00376)

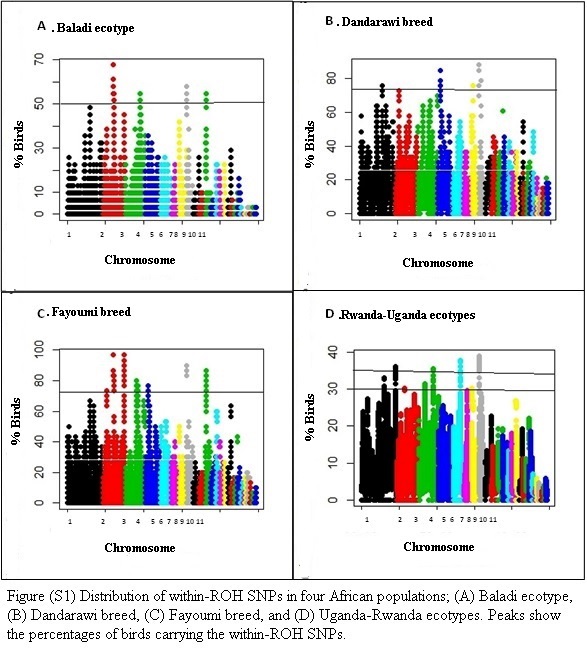

Supplement: Supplementary file 2 [file Image_1.JPEG]
